# Peripapillary retinal nerve fibre layer thinning in patients with X-linked retinoschisis

**DOI:** 10.1136/bmjophth-2024-001832

**Published:** 2024-08-29

**Authors:** Peter Kiraly, Ana Uršula Gavrić, Felix F Reichel, Johannes Birtel, Luca Mautone, Yevgeniya Atiskova, Philipp Herrmann, Martina Jarc-Vidmar, Marko Hawlina, Susan M Downes, M Dominik Fischer

**Affiliations:** 1 Oxford Eye Hospital, Oxford University Hospitals NHS Foundation Trust, Oxford, UK; 2 Nuffield Laboratory of Ophthalmology, Nuffield Department of Clinical Neurosciences, University of Oxford, Oxford, UK; 3 Eye Hospital, University Medical Centre Ljubljana, Ljubljana, Slovenia; 4 University Eye Hospital Tübingen, University of Tübingen, Tübingen, Germany; 5 Department of Ophthalmology, University Medical Center Hamburg-Eppendorf, Hamburg, Germany; 6 Department of Ophthalmology, University Hospital of Bonn, Bonn, Germany

**Keywords:** imaging, macula, retina, vision

## Abstract

**Aims:**

To assess peripapillary retinal nerve fibre layer (pRNFL) thickness in patients with X-linked retinoschisis (XLRS), as pRNFL thinning may limit functional improvements in gene therapy trials.

**Methods:**

This retrospective multicentre study included 49 eyes from 25 patients diagnosed with XLRS. Data collected with multimodal imaging at baseline and last follow-up (when available) included age, best-recorded visual acuity (BRVA), central retinal thickness (CRT), macular volume (MV), presence and location of peripheral retinoschisis and pRNFL thickness in the global (G), superotemporal (TS), superonasal (NS), inferotemporal (TI), inferonasal (NI), nasal (N) and temporal (T) sectors. Retinal sensitivity, assessed by microperimetry, was also recorded for seven patients at baseline.

**Results:**

pRNFL was thinner (below the fifth percentile) in at least one sector in 72% of the right eyes and 79% of the left eyes, with thinning across three or more sectors in 20% of the right and 17% of the left eyes. In 44% of cases, thinning occurred in the temporal sectors of both eyes, with no nasal sectoral thinning. Peripheral retinoschisis quadrants topographically matched the corresponding thinned pRNFL sectors. A strong positive correlation was found between MV and temporal pRNFL thickness (*r*=0.71, p<0.01), while weak negative correlation trends were noted with age (p=0.05) and BRVA (logMAR; p=0.12) related to temporal thickness of pRNFL sectors.

**Conclusion:**

pRNFL thinning, predominantly sectoral and linked to macular or peripheral retinoschisis, occurs in about three-quarters of patients with XLRS, while diffuse thinning occurs in one-fifth. Temporal pRNFL thinning might occur only after the collapse of intraretinal cystoid cavities in the macula.

**WHAT IS ALREADY KNOWN ON THIS TOPIC**

- Peripapillary retinal nerve fibre layer (pRNFL) thinning has been described in inherited retinal dystrophies, including X-linked retinoschisis (XLRS). pRNFL thinning could be a bottleneck for functional improvement in emerging XLRS gene therapy trials.

**WHAT THIS STUDY ADDS**

- pRNFL thinning in at least one sector occurred in 72% of the right eyes and 79% of the left eyes, while diffuse thinning occurred in 20% of the right eyes and 17% of the left eyes. Peripheral retinoschisis quadrants topographically matched the corresponding thinned pRNFL sectors. Smaller macular volume correlated strongly with temporal pRNFL thinning, while older age and worse best-recorded visual acuity showed trends towards temporal pRNFL thinning.

**HOW THIS STUDY MIGHT AFFECT RESEARCH, PRACTICE OR POLICY**

- Our study showed that pRNFL thinning in the temporal sector might occur after the collapse of intraretinal cystoid cavities. Therefore, pRNFL thickness might be preserved in young adults with XLRS, a period previously identified as the optimal timing for gene therapy.

## Introduction

 X-linked retinoschisis (XLRS) is a relatively common early-onset inherited retinal dystrophy (IRD), with prevalence estimated to range between 1 in 5000 and 1 in 20 000 in males.^1^ It is caused by mutations in the *RS1* gene, with more than 200 different pathogenic variants described, and the majority of these mutations leading to null alleles or non-functional proteins.^2 3^ This results in a deficient and/or non-functional retinoschisin-1 protein, which plays an important role in cell-to-cell adhesion and is mostly expressed in both photoreceptors and bipolar cells. Consequently, the typical XLRS phenotype is characterised by neurosensory retinal splitting within the macula in most, and peripheral retinoschisis in approximately half of the patients.^2^ Macular splitting is best visualised on optical coherence tomography (OCT) imaging as intraretinal cystoid cavities (ICCs), which are predominantly found in the inner nuclear layer.^4 5^ Approximately 20% of patients with XLRS develop complications such as retinal detachment and/or vitreous haemorrhage, which are associated with poor functional outcomes.^2 6^ Macular phenotype and visual acuity in patients with XLRS change with age. Patients usually present with ICCs and mild visual impairment in early childhood. In early adulthood, functional and morphological parameters can remain stable for several years before best-recorded visual acuity (BRVA) declines to moderate impairment. Ultimately, ICC collapse leads to retinal atrophy and severe vision loss.^6^

Visual acuity deterioration in patients with XLRS is primarily associated with macular changes.^7^ While total retinal thickness and BRVA only show a limited correlation,^6^ specific alterations in the outer retinal layers, such as photoreceptor outer segment thinning, disruption of the external limiting membrane and ellipsoid zone, are clearly associated with poor BRVA in patients with XLRS.^2^ Interestingly, optic disc pallor, known to be associated with BRVA worsening,^8^ was found in about 16% of patients in a large cohort of patients with XLRS.^2^ To date, only one study has evaluated the thickness of the peripapillary retinal nerve fibre layer (pRNFL) in 24 patients with XLRS, reporting that 62.5% of the patients exhibited pRNFL thinning in at least one quadrant of at least one eye.^9^ Therefore, the worsening of visual function might be associated not only with macular changes but also with optic nerve disc atrophy.

Until now, no effective treatment has been available for patients with XLRS.^6^ Some studies report that carbonic anhydrase inhibitors decrease ICC volume without clinically significant gains in visual acuity,^2 10^ while other studies have reported no morphological and/or functional benefits and spontaneous fluctuations in ICC volume.^6 11^ XLRS could be amenable to gene supplementation therapy as a monogenic disease linked to loss of function mutations in the *RS1* gene. Intravitreal gene therapy in *RS1* knockout mice demonstrated long-term retinal morphology rescue and ICC resolution,^12^ while human trials using intravitreal application of adeno-associated virus for XLRS showed good safety profile but no morphological or functional improvements.^13^ Another clinical trial is currently enrolling patients to test the safety and efficacy of subretinal gene therapy for XLRS.^6^

Any treatment for XLRS would need to prove functional efficacy, which might be limited due to optic nerve disc atrophy even after potential successful ICC resolution. Evaluating pRNFL thickness and its changes over time in patients with XLRS could help identify the potential or limitations for functional gains in these patients. In our study, we aimed to determine the symmetry of inter-eye pRNFL thickness, the presence and topographical pattern of pRNFL changes, the natural course of longitudinal pRNFL thickness measurements and the correlations of pRNFL thickness with age, as well as other morphological and functional parameters.

## Materials and methods

Data were retrospectively collected from several IRD centres: Oxford Eye Hospital; Department of Ophthalmology at University Medical Center Hamburg-Eppendorf; Department of Ophthalmology at University Hospital Bonn; and the University Eye Hospital Ljubljana. XLRS diagnosis was confirmed by an IRD specialist based on a comprehensive review of medical histories, slit-lamp biomicroscopy, multimodal imaging and full-field electroretinography (ERG). Genetic analysis was performed on all individuals, confirming the presence of pathogenic *RS1* mutations. Data were gathered at baseline and at the final follow-up (if applicable), including various parameters such as the pathogenic *RS1* sequence variants, patient age, BRVA, central retinal thickness (CRT), macular volume (MV) and pRNFL thickness analysis, as well as the presence and quadrants of peripheral retinoschisis.

BRVA measurements were conducted with Snellen charts, and the obtained values were then transformed into logMAR units for the purpose of statistical evaluation. Fundus images were obtained with an Optos device (Optomap P200, Optos plc, Dunfermline, UK). OCT for macular and pRNFL analysis was obtained with the Spectralis imaging platform (Heidelberg Engineering, Heidelberg, Germany). When severe retinal pathology resulted in incorrect automatic segmentation in macular or pRNFL scans, manual segmentation was performed by a medical retina specialist. After adjusting the segmentation if necessary, the imaging software automatically determined the CRT within a 1 mm diameter centred on the fovea, and the MV within a 6 mm diameter region. The pRNFL thickness was determined from the inner border of the internal limiting membrane to the inner layer of the ganglion cell layer. The thickness of the pRNFL was compared with the Spectralis pRNFL reference database, which is adjusted for age and specific to individuals of European descent. One eye that had unreliable retinal and pRNFL thickness measurements because of significant epiretinal membrane was omitted from the analysis. Mean global (G), superotemporal (TS), superonasal (NS), inferotemporal (TI), inferonasal (NI), nasal (N) and temporal (T) pRNFL thickness were assessed. Using the MP-3 device (NIDEK, Gamagori, Japan), retinal sensitivity was evaluated. The testing method included a 4–2 staircase strategy, projecting stimuli for 200 ms, and using a stimulus size equivalent to Goldmann III within a 12 degree area around the foveola.

Statistical analyses were conducted with SPSS Statistics for Windows, V.28 (IBM, Armonk, New York, USA), using a significance level of p<0.05 to establish statistical significance. The Pearson correlation coefficient was employed to evaluate correlations between both eyes, as well as between morphological and functional parameters within the same eye. The Wilcoxon signed-rank test was used to assess the statistical significance of the difference between pRNFL thickness at baseline and at the last follow-up.

Patients or the public were not involved in the design, conduct, reporting or dissemination plans of our research.

## Results

### Clinical, genetic characteristics and inter-eye pRNFL symmetry

Our study included 25 patients with XLRS with a median age of 27 years, ranging from 7 to 61 years at baseline. Follow-up was available for 11 patients with XLRS with a median duration of 33 months and ranging from 6 to 64 months. Peripheral retinoschisis in any of the quadrants was observed in 11 (44%) patients, with the most commonly affected area being the inferotemporal quadrant in 10 (40%) patients, followed by the inferonasal quadrant in 6 (24%), and the superotemporal quadrant in 5 (20%). No retinoschisis was observed in the superonasal quadrant in our cohort. Average BRVA (±SD) (logMAR) at presentation was 0.38 (±0.19) in the right eye and 0.43 (±0.21) in the left eye. All patients had intraocular pressure within normal limits and had no history of glaucoma. Average CRT (±SD) (µm) at presentation was 366 (±151) in the right eye and 360 (±152) in the left eye. Morphological parameters at baseline and inter-eye symmetry between both eyes are presented in table 1.

**Table 1:** Morphological parameters at baseline and inter-eye symmetry between both eyes

	MV (x̄±SD) mm³	G pRNFL(x̄±SD) µm	TS pRNFL(x̄±SD) µm	NS pRNFL(x̄±SD) µm	TI pRNFL(x̄±SD) µm	NI pRNFL(x̄±SD) µm	N pRNFL(x̄±SD) µm	T pRNFL(x̄±SD) µm
Right eye	8.32 (±1.91)	94 (±20)	128 (±32)	123 (±23)	123 (±28)	90 (±31)	82 (±24)	69 (±32)
Left eye	8.05 (±1.79)	94 (±13)	138 (±52)	128 (±21)	117 (±22)	95 (±21)	76 (±16)	61 (±13)
Inter-eye symmetry	r=0.74p<0.01	r=0.66p<0.01	r=0.81p=0.03	r=0.43p=0.03	r=0.70p<0.01	r=0.79p<0.01	r=0.59p<0.01	r=0.40p=0.05

G pRNFLglobal peripapillary retinal nerve fibre layer thicknessMVMacular volumeNI pRNFLinferonasal peripapillary retinal nerve fibre layer thicknessN pRNFLnasal peripapillary retinal nerve fibre layer thicknessNS pRNFLsuperonasal peripapillary retinal nerve fibre layer thicknesspstatistical significance between both eyes*r*Pearson correlation coefficientSDstandard deviationTI pRNFLinferotemporal peripapillary retinal nerve fibre layer thicknessT pRNFLtemporal peripapillary retinal nerve fibre layer thicknessTS pRNFLsuperotemporal peripapillary retinal nerve fibre layer thicknessx̄average

Genetic testing identified 16 pathogenic *RS1* mutations, with the most common being c.214G>A p.(Glu72Lys), observed in four patients. Microperimetry was conducted in seven patients with an average retinal sensitivity (±SD) of 22.1 (±4.9) dB in the right eye, and 21.6 (±7.0) dB in the left eye.

### pRNFL comparison to normative database and logitudinal pRNFL changes

**Figure 1:**
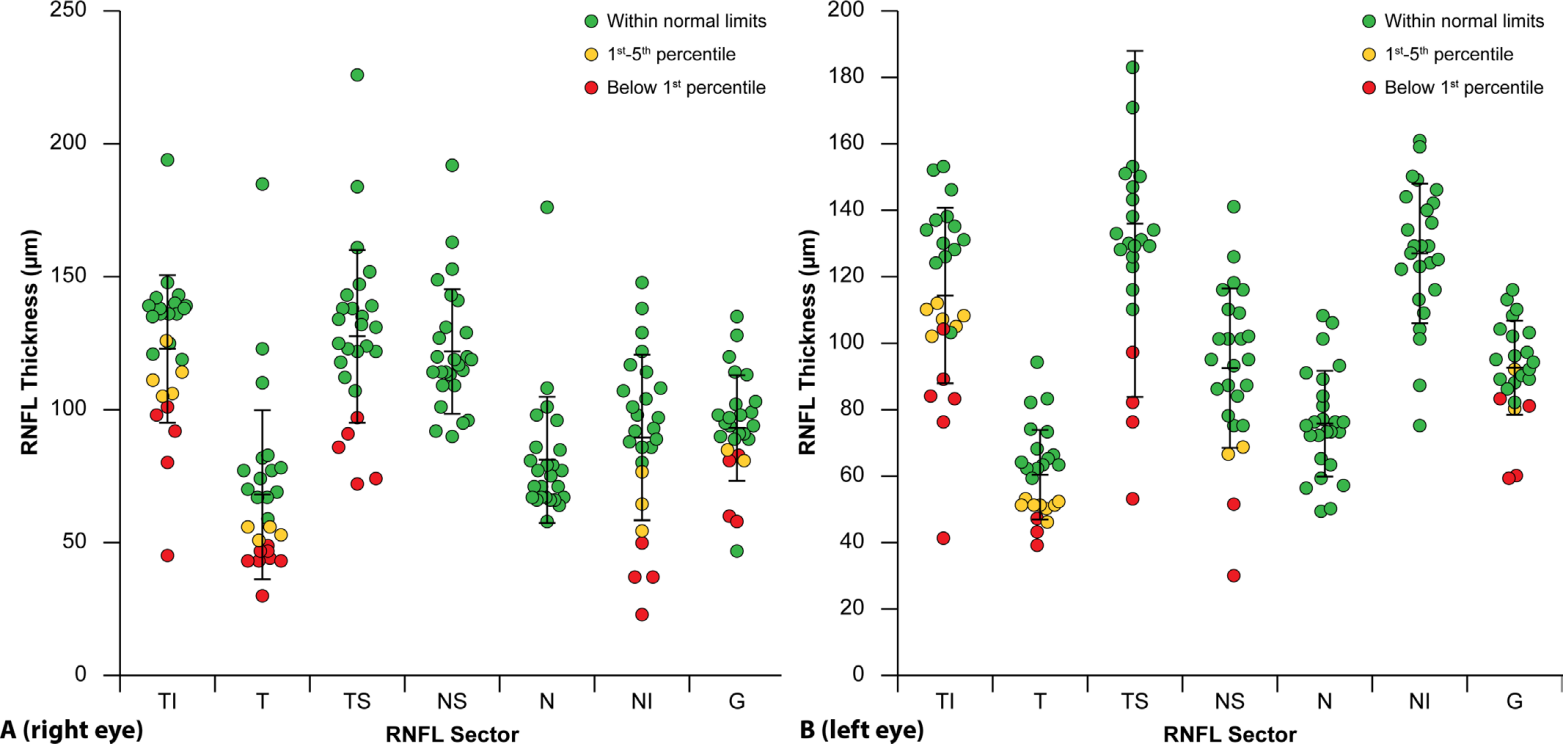
Peripapillary RNFL thickness in patients with X-linked retinoschisis and comparison with the normative reference database for the right eye (**A**) and the left eye (**B**). RNFL, retinal nerve fibre layer.

pRNFL was thinner (below the fifth percentile) in at least one sector in 18 (72%) of the right eyes, and in 19 (79%) of the left eyes (figure 1). One pRNFL sector was thinner in 9 (36%) right eyes and 8 (33%) left eyes; two sectors were thinner in 3 (12%) right eyes and 5 (21%) left eyes; three or more sectors were thinner in 5 (20%) right eyes and 4 (17%) left eyes. In 11 (44%) eyes, the pRNFL was thinner in the temporal part in both eyes, while no thinning was observed in the nasal part in any of the patients. Longitudinal changes in pRNFL thickness from baseline to the last follow-up were analysed only for the right eye in patients who had follow-up data (table 2). These data were available for 11 patients with XLRS, with a median follow-up duration of 33 months, a mean duration of 31 months and a range from 6 to 64 months.

**Table 2:** Longitudinal pRNFL thickness changes in the right eye from baseline to the last FU

	G pRNFL(x̄±SD) µm	TS pRNFL(x̄±SD) µm	NS pRNFL(x̄±SD) µm	TI pRNFL(x̄±SD) µm	NI pRNFL(x̄±SD) µm	N pRNFL(x̄±SD) µm	T pRNFL(x̄±SD) µm
Baseline	103 (±14)	135 (±36)	131 (±28)	136 (±10)	94 (±28)	90 (±32)	76 (±39)
Last FU	94 (±11)	133 (±20)	122 (±30)	127 (±21)	93 (±25)	81 (±22)	63 (±16)
P	0.06	0.82	0.28	0.14	0.51	0.20	0.22

FUfollow upG pRNFLGlobal peripapillary retinal nerve fibre layer thicknessNI pRNFLinferonasal peripapillary retinal nerve fibre layer thicknessN pRNFLnasal peripapillary retinal nerve fibre layer thicknessNS pRNFLsuperonasal peripapillary retinal nerve fibre layer thicknessPstatistical significance between longitudinal pRNFL measurementsSDstandard deviationTI pRNFLinferotemporal peripapillary retinal nerve fibre layer thicknessT pRNFLtemporal peripapillary retinal nerve fibre layer thicknessTS pRNFLsuperotemporal peripapillary retinal nerve fibre layer thicknessx̄average

### Correlations between pRNFL thickness, morphological characteristics, visual acuity and age

**Figure 2:**
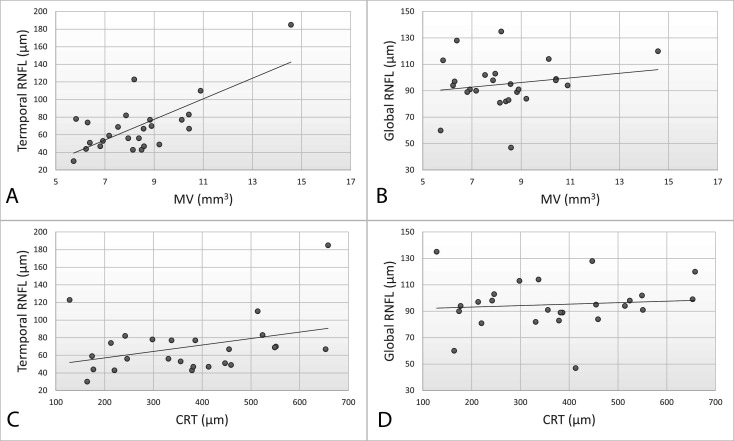
Correlations between pRNFL and retinal thickness in the right eyes of patients with X-linked retinoschisis. A strong positive correlation was observed between MV and temporal pRNFL (*r*=0.71, p<0.01) (**A**), and a negligible correlation between MV and global pRNFL (*r*=0.18, p=0.38) (**B**). A weak positive correlation between CRT and temporal pRNFL (*r*=0.37, p=0.06) (**C**) and a negligible correlation between CRT and global pRNFL (*r*=0.14, p=0.5) (D) were observed. CRT, central retinal thickness; MV, macular volume; pRNFL, peripapillary retinal nerve fibre layer thickness.

Correlations were calculated only for the right eye. No statistically significant correlations were observed between pRNFL and age; however, there was a negative trend between age and TS pRNFL (*r*=−0.38; p=0.05). Weak negative correlation was observed between BRVA (logMAR) and G pRNFL (*r*=−0.28, p=0.08); BRVA and TI pRNFL (*r*=−0.32, p=0.12); and BRVA and T pRNFL (*r*=−0.31, p=0.12). No statistically significant correlation was observed between CRT and any pRNFL sectors; however, a positive correlation trend was observed between CRT and T pRNFL (*r*=0.37, p=0.06). A strong correlation was observed between MV and T pRNFL (*r*=0.71, p<0.01), while no other correlation between MV and other sectors was statistically significant (figure 2). In the subset of patients (n=7) with microperimetry data, no statistically significant correlations were observed between G pRNFL, T pRNFL, and the mean retinal sensitivity.

## Discussion

Our study shows that the pRNFL was thinner in at least one segment in approximately three-quarters of the eyes, while diffuse pRNFL thinning across three or more sectors was observed in about one-fifth of the eyes. pRNFL thinning was predominantly seen in the temporal part, while the nasal pRNFL thickness was within normal limits. In 11 patients with XLRS with a relatively short follow-up period (median=33 months, range=6–64 months), longitudinal changes in pRNFL thickness showed non-statistically significant thinning across all sectors. Inter-eye symmetry showed moderate to strong correlations across all sectors, except for the temporal sector, which had a weak correlation. Regarding correlation analysis, older age and worse BRVA showed trends with thinning in temporal pRNFL sectors, and smaller MV showed a strong correlation with the T pRNFL thinning.

In several IRDs, a thinner pRNFL has been reported, which could result from anterograde axonal degeneration.^914^,^16^ This type of degeneration, referred to as 'dying forward,' involves the impairment of a subsequent neuron due to issues with a preceding neuron, such as impaired afferent innervation or decreased transmission of growth and survival factors.^17 18^ Mutations in the *RS1* gene lead to a lack of functioning retinoschisin, which in turn leads to synaptic dysfunction between photoreceptors and bipolar cells due to the mislocalisation of intracellular proteins in the postsynaptic structure.^19^ This results in a hallmark feature of XLRS, the electronegative ERG: a nearly normal a-wave, indicating photoreceptor activation, leads to a characteristically weak b-wave response, which reflects synaptic dysfunction affecting the bipolar cell population.^20^ Although the retinoschisin protein is most abundantly expressed in the outer retina, it is also synthesised locally in the inner retina.^19^ It is not clear whether the lack of RS1 in ganglion cells has a direct impact on pRNFL thickness.

Macular changes (schisis/atrophy) are reported in the majority of patients with XLRS, and about half of these patients also have peripheral retinoschisis.^6^ In the macula, ICCs are most commonly observed in the inner nuclear layer (79%), while in the periphery, they are commonly observed in the ganglion cell layer, which can lead to bullous retinoschisis.^21^ Senile retinoschisis is known to cause absolute scotoma,^22^ and visual field loss is associated with corresponding sectoral pRNFL thinning.^23^ Therefore, significant pRNFL thinning in patients with XLRS might be observed in sectors affected by current or past retinoschisis. In our patients with XLRS, pRNFL thinning was observed in at least one sector in about three-quarters of the patients, with one to two sectors affected in about two-thirds of these cases, indicating that pRNFL thinning in patients with XLRS is mostly sectoral. However, diffuse pRNFL thinning, with more than three sectors affected, was observed in about one-fifth of all patients, which closely resembles the percentage (15.8%) of patients with XLRS reported to have optic disc pallor.^2^ In a retinal organoid model of XLRS from a patient with the c.625C/T (p.Arg209Cys) variant in the *RS1* gene, a progressive loss of *OPA1* gene expression was observed, with *OPA1* mutations being the most common genetic cause of autosomal dominant optic atrophy.^2 24^ Thus, some *RS1* pathogenic variants in patients with XLRS might be associated with decreased *OPA1* gene expression, which could explain diffuse RNFL thinning in some patients. Our sample size of patients with XLRS was too small to observe correlations between pRNFL thinning and genotype. Further studies involving more patients with XLRS, and/or retinal organoid models with various *RS1* pathogenic variants, are needed to confirm a possible additional mechanism of pRNFL thinning that is independent from retinoschisis. Our results showed moderate to strong inter-eye correlations between pRNFL sectors, which would be important in potential gene therapy trials where an untreated eye could serve as a control. Regarding longitudinal pRNFL thickness changes, non-statistically significant thinning was observed in all sectors, which could be explained by a relatively short follow-up period (median=33 months, range=6–64 months) and the expected pRNFL thinning that occurs with age. The wide range of follow-up period durations could also influence the consistency of the longitudinal changes observed.

**Figure 3:**
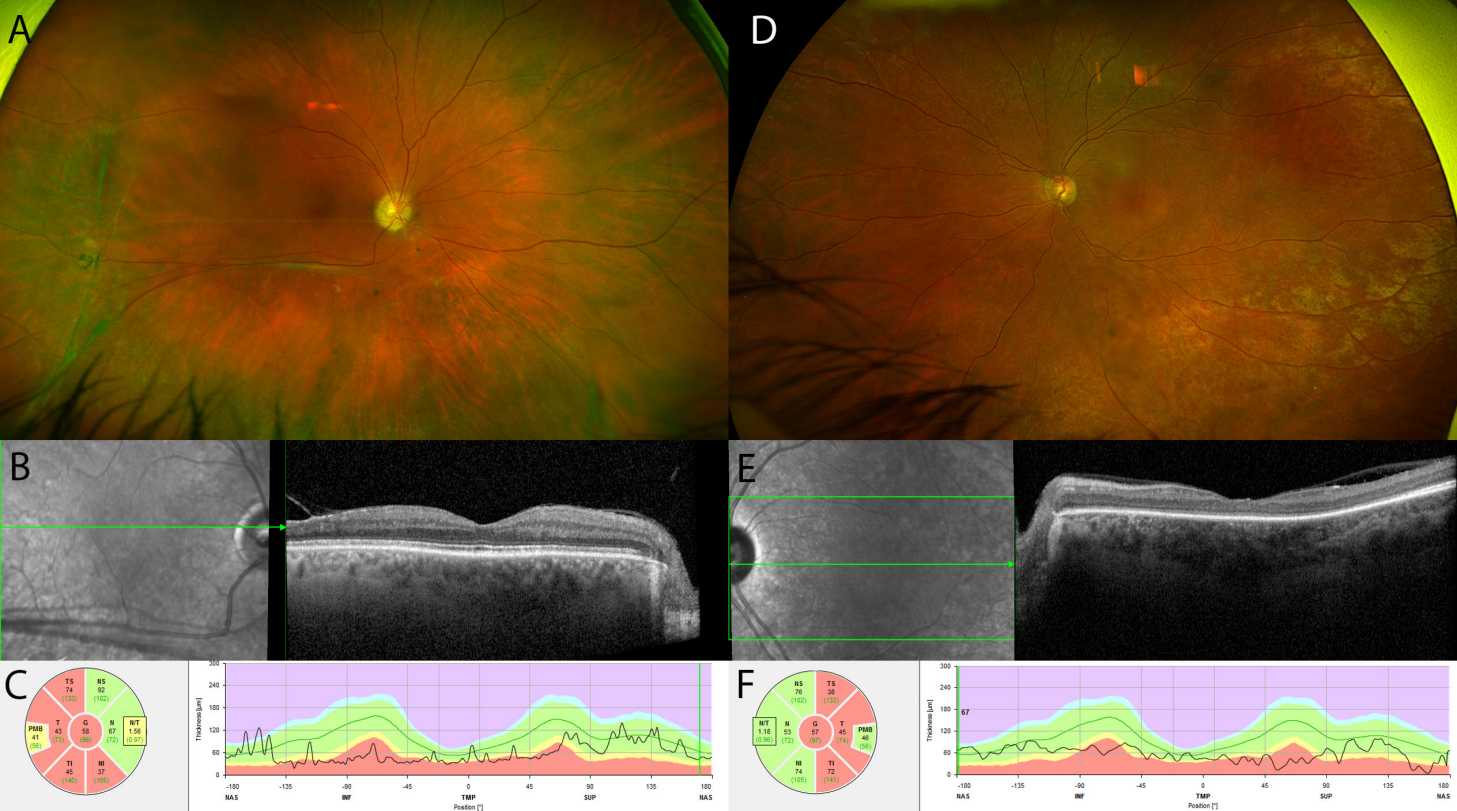
RNFL thinning can occur due to macular or peripheral retinoschisis. A patient with XLRS with extensive peripheral retinoschisis (**A**), near-normal macular structure (**B**) and significantly decreased RNFL thickness (**C**). Another patient with XLRS exhibits no peripheral retinoschisis (**D**), macular thinning following the collapse of intraretinal cystoid cavities (**E**) and temporal RNFL thinning. RNFL, retinal nerve fibre layer; XLRS, X-linked retinoschisis.

In the right eye, peripheral retinoschisis quadrants topographically matched the corresponding thinned pRNFL sectors. Ten eyes had retinoschisis and pRNFL thinning in the inferotemporal, five in the superotemporal, six in the inferonasal and none in the superonasal quadrant/sector. These results clearly suggest that peripheral retinoschisis leads to pRNFL thinning in the corresponding sectors. This can also be seen in figure 3A–C, which shows a patient with near-normal macular structure, extensive peripheral retinoschisis and pRNFL thinning in sectors corresponding to the retinoschisis. In contrast, nearly all (24 out of 25) patients with XLRS exhibited maculopathy, while only 11 out of 25 showed pRNFL thinning in the temporal region, which includes the papillomacular bundle. No pRNFL thinning was observed in the nasal sector. The most likely explanation is that macular retinoschisis is milder than peripheral retinoschisis, involving only ICC rather than a complete separation of retinal layers. Interestingly, higher MV measurements strongly correlated with higher pRNFL in the temporal sector. MV correlated more strongly with T pRNFL than with CRT because it covers a larger area within the macula. Additionally, MV demonstrated a stronger correlation with T pRNFL than with G pRNFL, due to its closer correspondence with the retinal topographical area. In patients with XLRS, maculopathy changes with age. Extensive ICC volume is present in the majority of patients during childhood, followed by a collapse of ICC and corresponding MV reduction in middle-aged to older patients with XLRS.^6^ Consequently, younger patients with higher MV volume maintain preserved pRNFL thickness, while older patients with reduced MV exhibit pRNFL thinning in the temporal sector. This is illustrated in the online supplemental figure 1, where a younger patient with XLRS with a high MV and extensive ICC volume has pRNFL thickness within normal limits. In contrast, figure 3D–F depicts an older patient with XLRS with no peripheral retinoschisis, collapsed ICC, reduced macular thickness and significant pRNFL thinning in the temporal sectors. Although no statistically significant correlations were observed between pRNFL and age, a negative correlation was noted between age and TS pRNFL. Our results suggest that pRNFL thinning related to maculopathy occurs only after the collapse of ICC and subsequent MV thinning. This could have important implications for patient selection in emerging XLRS gene therapy trials, as pRNFL thinning could be a bottleneck for functional improvement. However, we showed that T pRNFL thickness is still within normal limits in patients with XLRS with high MV, suggesting good potential for vision gain in this subgroup.^2^

Previous studies showed that BRVA worsening correlated with macular atrophy in the outer retinal layers.^2 6 7^ However, a hypothesis from a previous paper suggests that pRNFL thinning may also contribute to vision loss.^7^ BRVA in patients with XLRS is impaired from early childhood,^2^ and macular ICCs are observed in very young children.^25^ Moreover, an *RS1* knockout mouse model revealed photoreceptor apoptosis starting soon after birth.^26^ Our data show that pRNFL is thicker and within normal limits when MV is higher, which is typically the case in younger patients. Additionally, in patients with optic atrophy, mean global pRNFL thickness correlated with visual field loss but not with visual acuity.^27^ Our data suggest that BRVA might not be impaired due to pRNFL thinning early in life and that pRNFL thinning occurs secondary to other retinal morphological changes. The online supplemental figure 1 illustrates a patient with XLRS with reduced retinal sensitivity in the topographical areas of ICC, while the outer retinal layers are largely intact and pRNFL thickness is within normal limits. This also indicates that visual function worsening early in life might not be related to pRNFL thinning. However, further studies on ganglion cell complex thickness and retinal sensitivity are needed to estimate the pattern of visual loss at different stages of XLRS.

A limitation of this study is the relatively small sample size of 25 patients. Future studies with larger sample sizes would be needed to validate and extend the findings.

In conclusion, our results show that pRNFL thinning is observed in about three-quarters of patients with XLRS. pRNFL thinning is mostly sectoral and might occur secondary to macular or peripheral retinoschisis. However, in some patients, pRNFL thinning is diffuse and may be associated with specific *RS1* pathogenic variants leading to optic atrophy independent of retinoschisis. pRNFL thinning in the temporal sector might occur in patients with XLRS after the collapse of ICC and atrophy of the outer retinal layers, which has important implications for XLRS gene therapy trials. BRVA worsening in patients with XLRS is mostly attributable to ICC, outer retinal atrophy and known complications associated with XLRS (retinal detachment, vitreous haemorrhage), rather than to pRNFL thinning.

## supplementary material

10.1136/bmjophth-2024-001832 online supplemental figure 1

## Data Availability

Data are available upon reasonable request.
