# Supplementary material for: Peripapillary retinal nerve fibre layer thinning in patients with X-linked retinoschisis
Source: BMJ Open Ophthalmol. 2024 Aug 29;9(1):e001832. doi: 10.1136/bmjophth-2024-001832 (PMC11367349; doi:10.1136/bmjophth-2024-001832)
Supplement: online supplemental figure 1 [file bmjophth-9-1-s001.pdf]

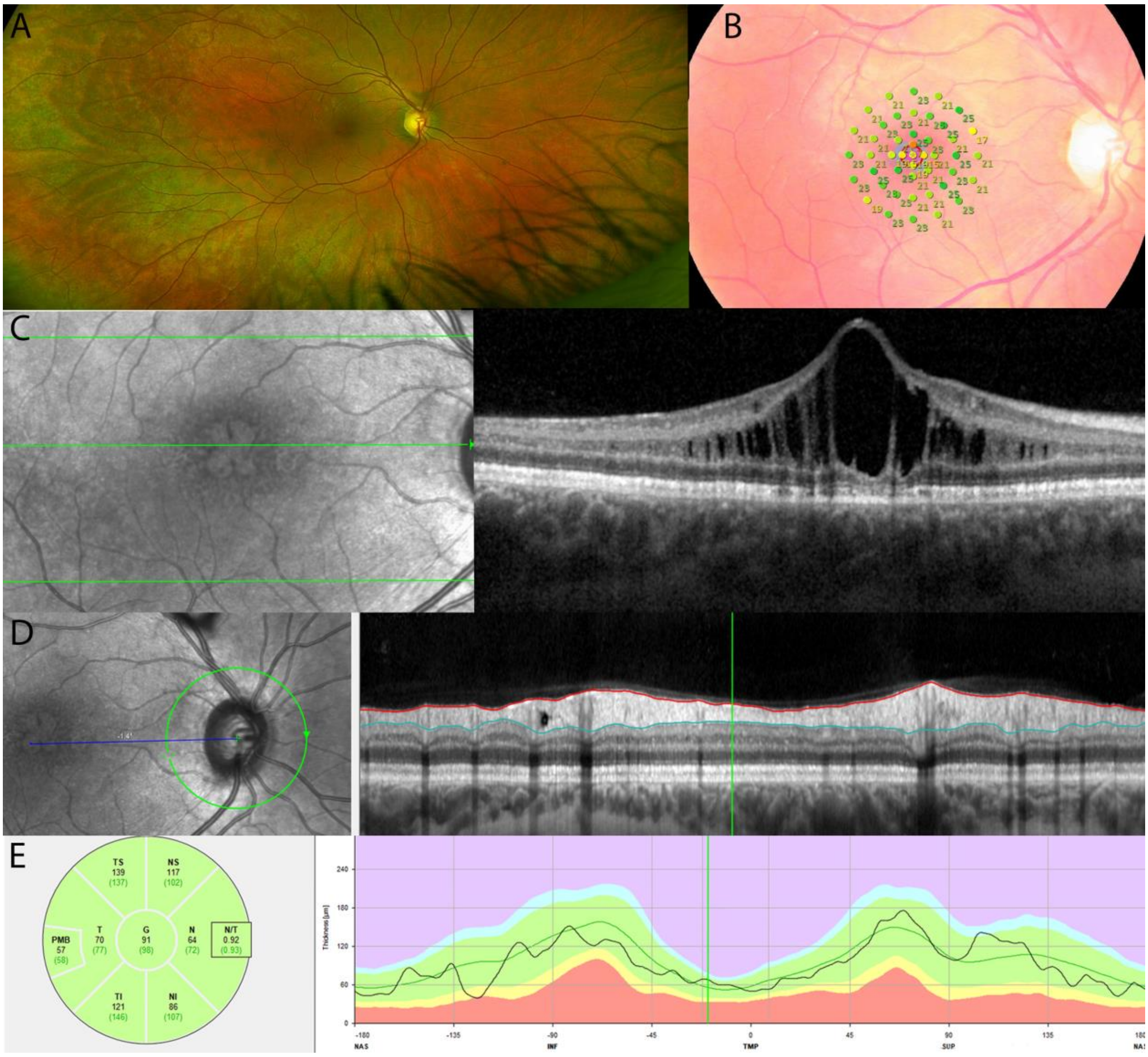

Supplemental Figure 1. Multimodal imaging in a patient with X-linked retinoschisis in his late 20s. Fundus examination and Optos imaging showed no peripheral retinoschisis (A). Microperimetry revealed reduced retinal sensitivity (B) in the topographical areas with intraretinal cystoid cavities as observed on optical coherence tomography (OCT) (C), while peripapillary retinal nerve fiber layer (pRNFL) thickness was within normal limits (D, E).
